# Determinants of Anemia in Schoolchildren in the Highland Bolivia

**DOI:** 10.3390/microorganisms12122491

**Published:** 2024-12-03

**Authors:** Washington R. Cuna, Ivonne Contreras, Armando Rodriguez, Roberto Passera, Celeste Rodriguez

**Affiliations:** 1 Unidad de Inmunología Parasitaria, Facultad de Medicina, Universidad Mayor de San Andrés, La Paz 10077, Bolivia; washingtoncuna@gmail.com (W.R.C.); civcont@gmail.com (I.C.); arjoroze@gmail.com (A.R.); 2 Department of Medical Sciences, University of Torino, 10126 Torino, Italy; passera.roberto@gmail.com

**Keywords:** children, anemia, protozoa, helminths, iron deficiency anemia, anemia of inflammation

## Abstract

Anemia is a health problem of concern among schoolchildren in underprivileged rural regions, where recurrent parasitic infections are common. A cross-sectional study was conducted in 229 schoolchildren in rural highland Bolivia in the department of La Paz, an area with a high prevalence of protozoan and helminth infections, to determine the types and mechanisms of anemia. A substantial proportion of children (40.2%) were found to be anemic based on hemoglobin measurements. No associations were found between low hemoglobin levels and helminth or protozoan infections when evaluating infectious causes of anemia, nor with *Giardia lamblia* or *Blastocystis hominis*, which are associated with iron deficiency and nutrient malabsorption and were highly prevalent in this study. The significant association between anemia and hypochromia suggests iron deficiency, aligned with low hemoglobin levels. A total of 39 out of 150 children (26%) had markers consistent with iron deficiency anemia (IDA), 26 out of 127 children (20%) met the criteria for anemia of inflammation (AI). Furthermore, 12 of the 127 tested children (9.4%) met the criteria for mixed AI with IDA according to the soluble transferrin receptor (sTfR)/log ferritin levels, which increased significantly due to overall infections by *Hymenolepis nana* and *Ascaris lumbricoides* helminths. The findings highlight the need for integrated public health interventions to address iron nutrition and parasitic infections to effectively prevent anemia in this vulnerable population.

## 1. Introduction

Anemia is a global public health problem that affects approximately one third of the world’s population, mainly in developing countries [1], although pregnant women and preschool children are the populations most vulnerable to anemia due to reduced oxygen supply at a time when metabolic needs are high, anemia in schoolchildren is also of concern due to its impact on their physical and cognitive development [2,3], predisposing them to a higher frequency of morbidity [4,5,6]. It is generally recognized that anemia is one of the consequences of iron deficiency due to low dietary intake and iron-deficiency anemia (IDA) has been associated with significantly lower scores of on cognitive tests and significantly lower achievements than in non-anemic children [7]. In addition to IDA, another type of anemia, includes anemia of chronic disease, also termed anemia of inflammation (AI); these are the two most prevalent forms of anemia [8]. AI ranks second; its actual prevalence is difficult to assess as it often coexists with IDA. Iron-restricted erythropoiesis is part of the pathophysiology of both AI and IDA, making differential diagnosis difficult when both diseases coexist [9,10]. IDA and AI often affect the same individuals, especially in low-income countries with a high burden of parasitic diseases, where chronic blood loss and iron deficiency emerge due to helminth infestations (e.g., hookworms, *Trichuris trichiura*) [11,12,13]. The combination of serum transferrin receptor (sTfR) and ferritin measurements for the calculation of the sTfR/Ferritin index has been shown to accurately determine whether the occurrence of IDA, AI, or a combination of these mechanisms is involved in anemia, particularly in anemia with active inflammation [8]. Polyparasitism of concurrent helminth and protozoan parasites and a strongly increased risk of anemia are observed in schoolchildren from rural Bolivian altiplano (highland) regions, where clean water, adequate housing and sewage systems are not available [14]. This study builds on our previous research by examining the most likely mechanisms and biomarkers associated with anemia in the highland regions. Therefore, the aim of this study was to use more comprehensive laboratory testing in the differential diagnosis of various types of anemia among schoolchildren. To our knowledge, there are no studies in the literature evaluating the sTfR-Ferritin index which should be considered in the management of regional childhood anemia in this underprivileged area of Bolivia, in which recurrent gastrointestinal protozoa and helminth infections are endemic. 

## 2. Materials and Methods

### 2.1. Study Area, Population, and Assessment

Cross-sectional data were collected from schoolchildren of the Bolivian highland, where helminths and protozoan gastrointestinal parasites are known to be endemic. Surveys were carried out in different school units located at an altitude between 3.810 and 4.050 m above sea level, in which health and basic service conditions are regular to inappropriate; 55% have a water pipe supply, barely 30% have a flush toilet or a latrine, and most (99%) of the households lack piped sewers. A total of 229 children aged 5–13 years who participated in this study had blood drawn for anemia testing. Anthropometric measurements (i.e., height, weight) to assess the growth of children, were taken by trained local nurses following standardized procedures. The study received approval from the ethics committee of the Universidad Mayor de San Andres.

### 2.2. Diagnosis of Gastrointestinal Parasites

Stool samples were analyzed microscopically, samples under high suspicion for amebic dysentery (that is, bloody diarrhea with mucus) were carefully examined in fresh and in smears fixed and stained with Wright’s stain, a portion of the other samples was analyzed through Ritchie’s formol-ether concentration technique after Wright staining. Samples with eggs, larvae, or cysts, were considered positive for a species of parasite.

### 2.3. Testing for Anemia

Venous whole blood (5 mL) was collected via venipuncture and divided: 1 mL in a tube containing EDTA for complete blood count and 4 mL in a tube for serum preparation. The serum was frozen and used later for ferritin and sTfR measurements. In the highlands, cases of anemia with hemoglobin (Hb) levels < 14.4 g/dL corresponded to World Health Organization levels [15]; these levels together with serum ferritin (SF) < 30 ng/mL defined IDA. Anemia of inflammation was identified in children with SF levels 30–100 ng/mL and an sTfR/log SF ratio of less than 1. Cases of combined AI with IDA included samples with SF 30–100 ng/mL, and an sTfR/log ferritin ratio > 2 [8]. Ferritin and sTfR were assayed using commercial enzyme immunoassays (Monobind Inc., Lake Forest, CA, USA). Initially, all participants underwent hemoglobin measurements on-site by point of care testing using a HemoCue analyzer (Angelholm, Sweden). 

### 2.4. Statistical Data Analysis

Categorical variables were described as absolute and relative frequencies, while continuous ones as the median and inter quartile range (IQR). Non-parametric inferential analysis for categorical and continuous covariates was performed using Fisher’s exact test and the Mann–Whitney and Kruskal–Wallis ones, respectively. All *p* values were obtained via the two-sided exact method at the conventional 5% significance level. Data were analyzed in October 2024 by R 4.4.1 (R Foundation for Statistical Computing, Vienna, Austria. https://www.r-project.org).

## 3. Results

### 3.1. Study Population

Demographic characteristics and prevalence of helminth and protozoan parasite infections for 229 participants are shown in Table 1. There was a fairly balanced gender distribution with a slight majority of female participants (55%) compared to males (45%). Over 70% and 20% of children were infected with protozoan and helminth parasites, respectively. A substantial proportion of participants (40.2%) were anemic (90/229), indicating a significant public health problem. The percentages of malnutrition indicators such as stunting (4.8%), wasting (3.5%), and malnourished children (3.0%) were relatively low, suggesting that severe malnutrition is not widespread in this population. In general, there were no significant differences in the prevalence of sex, age, infection, malnutrition, stunting, wasting, or anemia as detected through point-of-care testing during the surveys.

### 3.2. Prevalence of Parasitic Infections

**Figure 1:**
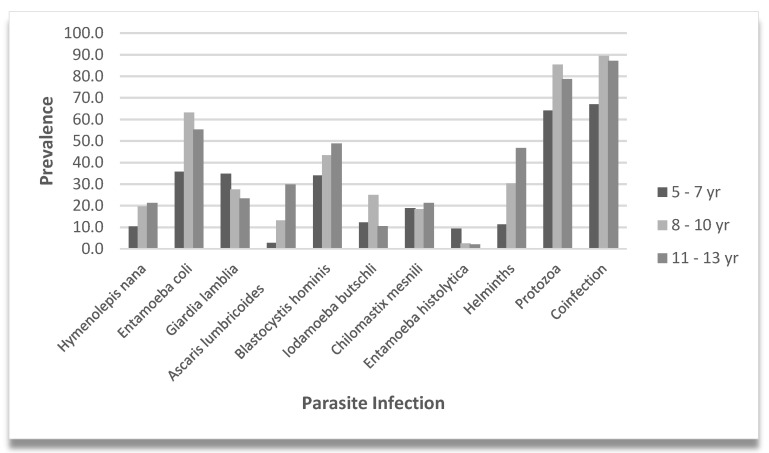
Parasite prevalence by age group. Vertical bars indicate the prevalence of specific parasite infections, helminth, protozoa, and coinfections. Percentages are shown for the 5–7, 8–10, and 11–13 year subgroups.

Figure 1 shows the prevalence of parasitic, protozoa, and helminth infections, and coinfections (protozoa and helminths) among children grouped by three age categories, 5–7, 8–10, and 11–13 years. Consistent with an earlier set up, the division of age into three age groups was defined in our previous study [14]. The data on parasitic infections highlighted a high prevalence of protozoan infections (74.2%), particularly *Entamoeba coli* (48.9%) and *Blastocystis hominis* (40.2%). *Entamoeba coli* mature cysts present up to eight nuclei and *E. histolytica/dispar* four. Microscopic identification of *E. histolytica* hematophagous trophozoites was accomplished using fresh and fixed stool samples. As previously reported, hematophagy is considered a distinguishing microscopic criterion for identifying *E. histolytica* [16,17,18]. Specific parasitic and helminth infections increased significantly with age. Across the age groups of 5–7-, 8–10-, and 11–13-years, the prevalence of each infection and polyparasitism increased significantly. There was a progressive increase in *H. nana* (99% CI, 0.031 to 0.041, *p* = 0.036), *A. lumbricoides* (*p* < 0.001), *B. hominis* (99% CI, 0.038 to 0.048, *p* = 0.043), and helminths (*p* < 0.001). There was a statistically significant association in the *E. coli* (*p* < 0.001), protozoan (*p =* 0.004), and coinfection (*p* < 0.001) infection rates, across age groups, particularly showing higher positivity in children over the age of 7 years. 

### 3.3. Types of Anemia and Iron Biomarkers Across Age Groups

Table 2 details data on hemoglobin levels, anemia status, and iron biomarkers among children classified as 5–7, 8–10, and 11–13 years of age. Hemoglobin measurements performed in venous blood revealed that 40.2% of children were anemic (hemoglobin < 14.4 g/dL). The percentage of children who were anemic was quite high across all age groups, with a slight decrease in 8–10-year-olds (36.0%) compared to 5–7 (43.1%) and 11–13-year-olds (40.4%) (Table 2).

**Figure 2:**
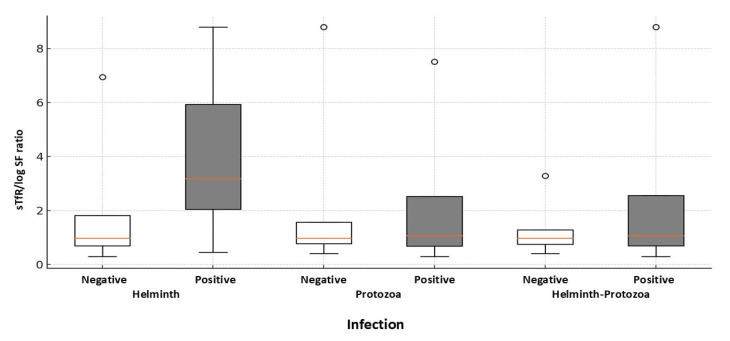
Boxplot comparing the soluble transferrin receptor (sTfR) to log ferritin ratios, defining combined anemia of inflammation with iron-deficiency anemia among study children with and without detectable infections. The distributions of sTfR/log SF ratios are presented for sTfR-tested children who either have (N = 12) or do not have (N = 127) helminth infections. The *p*-value for group differences is 0.001, determined via the Mann–Whitney U test.

Median serum ferritin levels varied slightly between age groups, with values ranging from 16.0 to 18.82 ng/mL and an overall median level of 17.27 ng/mL for all children. Median MCV measurements to determine the underlying cause of anemia (for example, nutritional deficiencies), fell within normal ranges (84.2 to 84.5 fL) in 120 of 225 (53%) cases, after accounting for four missing values; however, red cell macrocytosis (elevated MCV) was observed in 89 of 225 (39%) children. MCHC levels showed a small decrease in the median levels with increasing age, from 32.7 g/dL in the youngest group to 32.4 g/dL in the oldest group. The prevalence of hypochromasia (lower MCHC) increased with age, from 25.5% in children aged 5–7 years to 36.2% in those aged 11–13 years, being significantly higher (*p* = 0.006) among anemic children (36.7%) compared to those with normal hemoglobin status (21.6%). The 8–10-year group showed a significant increase (*p* < 0.001) in the median level of sTfR (2.35 mg/L) compared to the other groups, which had median values of 1.26 mg/L (5–7 years) and 2.18 mg/L (11–13 years). This is reflected in the higher sTfR/log ferritin ratio in the 8–10 years age group, with a median of 2.17 compared to the other groups (0.98, 0.70); however, the observed median differences were not statistically significant (*p* = 0.09). Detailed percentages of children classified as iron-deficient and anemic (26%), meeting the AI criteria (20.5%), and a smaller percentage (9.4%) characterized as mixed AI with IDA, are shown (Table 2). Reflecting deficient iron levels in our data, we observed a significant association between anemia and IDA, characterized by low levels of hemoglobin and ferritin (*p* > 0.001). Figure 2 presents box plots that compare the sTfR/log ferritin ratio in different types of infection (helminth, protozoa, and helminth–protozoa), with each category divided into negative and positive infection statuses. The positive groups show a wider range of sTfR/log ferritin ratio, suggesting greater variability in iron status or inflammatory response resulting from infection. In particular, children who are positive for helminth infections show a much higher median sTfR/log ferritin ratio compared to those who are negative. Children infected with *H. nana* and *A. lumbrioides* exhibited a significantly higher median sTfR/log SF value of 3.18 (range 2.04–5.93), compared to 0.97 (range 0.69–1.81) in uninfected children (*p* < 0.001). Mixed AI with IDA is a relatively rare condition in our study, observed only in 9.4% of cases, after accounting for missing data (102 cases, or 44.5%).

## 4. Discussion

The findings of this study conducted in the highlands of Bolivia indicate a substantial prevalence of anemia (40%) among schoolchildren in a rural area where protozoan infections predominate, followed by helminth infections [14,19]. While evaluating potential infectious causes of anemia, no statistically significant associations were found with helminth, or protozoan infections, despite a notable, progressive increase in the prevalence of these infections with age. Furthermore, we did not observe a statistically significant correlation between lower hemoglobin levels (<14.4 g/dL) and the presence of *G. lamblia* or *B. hominis,* protozoa known to be associated with iron and nutrient malabsorption, malnutrition, and highly prevalent in this survey [20,21,22]. Specifically, the capacity of *G. lamblia* to lower hemoglobin levels may be associated with its tendency to cause vitamin B12 and folic acid deficiencies, which could explain the macrocytosis (40%) observed in our study in the context of anemia [23,24]. Whether these metabolic alterations persist in our setting remains to be confirmed. The occurrence of macrocytosis and hypochromasia (27.7%) suggests overlapping causes for our observations in addition to deficiencies. The significant association between anemia and hypochromasia could be an indicator of iron deficiency reflecting lower hemoglobin levels.

The lower rates of malnutrition observed suggested that anemia was driven by factors other than malnutrition, likely, micronutrient deficiencies. The availability of iron rich food, such as meat, is limited, and children’s diet consists mainly of carbohydrates, vegetables, and fruits. The absence of heme iron from meat, a primary source of iron that is more easily absorbed by the body compared to non-heme iron from plant-based sources, along with increased vulnerability to inhibitors such as phytates and polyphenols, further hinders iron absorption [25]. Consequently, a significant proportion of anemia cases may be due to nutritional deficiencies, particularly a lack of dietary iron. Our findings indicate that 26% of anemic children had markers compatible with iron deficiency, including low hemoglobin and ferritin levels. This suggests that IDA together with true iron deficiency, are the main contributors to childhood anemia in the highlands of Bolivia. This assumption is further supported by a significant association between anemia and IDA. At the same time, 20% of anemic children with normal or moderately elevated ferritin levels, met the criteria for anemia of inflammation, suggesting that both altered iron status and coexisting inflammatory processes play a role in the development of anemia in our study. In the context of chronic inflammation, persistent parasitic infections have been found to impair the body’s ability to absorb dietary iron by promoting ferroportin degradation, thus reducing the use of existing iron stores for erythropoiesis [26,27,28]. Ferroportin internalization and degradation are mediated by the regulatory protein hepcidin which is up-regulated by interleukin (IL) 6 during inflammation [29,30]. This process decreases blood iron levels and leads to AI.

Significantly higher levels of sTfR in relation to serum ferritin, in our study, underscore the impact of IDA and AI as additional causes of anemia in 9.4% of highland children, and the effects of combined infections by helminths of *H. nana* and *A. lumbricoides* lead to mixed types of anemia, likely due to a variety of symptoms, including nutritional malabsorption, malnutrition, gastrointestinal bleeding, and iron deficiency attributed to these helminths [31,32,33,34].

There were strengths and weaknesses in our study. The use of comprehensive laboratory tests enabled the differentiation between IDA, AI, and mixed forms of anemia for this region with chronic infections, where overlapped symptoms could complicate diagnosis. However, due to budget limitations, we were unable to measure ferritin and sTfR levels for all children, which may have hindered an accurate representation of the impact of combined types of anemia in our setting. Analyzing only a single stool sample per child may lead to an underestimation of the true prevalence of parasitic infections. Furthermore, there is a lack of data on the intensity of helminth infections since health is intensity-related. Moreover, data on socioeconomic status, such as family size, income, and education level, which may contribute to inadequate nutrition linked to anemia and IDA, is lacking.

The high prevalence of anemia among children in highland areas is a significant public health concern, given its impact on childhood cognitive development and physical fitness. By identifying the underlying causes and the most impactful parasitic infections, this study provides actionable insights to help reduce the burden of anemia in this vulnerable population and in similar settings across different regions, particularly in high-altitude, low-income areas. The specific parasite patterns and the diagnostic approach such as the use of sTfR/log ferritin ratios provide unique information into anemia in the Bolivian highlands which may differ in some aspects from the more acute or geographically distinct forms of anemia seen in malaria or schistosomiasis [27,35,36]. Therefore, this study enhances the global understanding of how various parasites contribute to anemia and provides insights into the regional epidemiology of the condition.

Our findings highlight key challenges that need to be addressed to improve childhood health conditions. These include the need for integrated public health interventions, such as enhanced regular deworming and sanitation to prevent recurrent infections, as well as improved iron nutrition to effectively combat anemia in this vulnerable population.

## Figures and Tables

**Table 1:** Demographic characteristics of participants and parasitic infections for the anemia study in the highlands of Bolivia.

Characteristic	Highlands (N = 229)
Sex (%)	
Female	126 (55)
Male	103 (45)
Age (%)	
5–7	106 (46.3)
8–10	76 (33.2)
11–13	47 (20.5)
Helminths (%)	57 (24.9)
Protozoa (%)	170 (74.2)
Helminths-protozoa (%)	180 (78.6)
Anemic * (%)	90 (40.2)
Malnourished † (%)	7 (3.0)
Stunted ‡ (%)	11 (4.8)
Wasted § (%)	8 (3.5)

* Hemoglobin < 14.4 g/dL. † Weight for hight z score (WAZ) < −2. ‡ Height for age z score (HAZ) < −2. § Body mass index (BMI) for age z score (BAZ) < −2.

**Table 2:** Anemia and anemia biomarkers by age group among the highland children of Bolivia.

Variable	5- to 7-Year-Olds	8- to 10-Year-Olds	11- to 13-Year-Olds	All Children
Hemoglobin in g/dL, median (IQR)	14.5 (13.8–15.4)	14.6 (13.5–14.8)	14.3 (14.0–15.2)	14.6 (13.8–15.2)
Percent anemic	43.1	36.0	40.4	40.2
MCV in fL median (IQR)	84.2 (80.4–90.1)	84.5 (78.8–90.3)	84.2 (75.6–88.1)	84.2 (80.3–90.1)
Percent macrocytosis	43.1	41.3	29.8	40.0
MCHC in g/dL median (IQR)	32.7 (32.0–33.5)	32.6 (31.8–33.3)	32.4 (31.6–33.0)	32.6 (31.9–33.3)
Percent hypochromasia	25.5	29.0	36.2	27.7
Serum ferritin in ng/mL, median (IQR)	17.8 (10.3–30.8)	16.0 (9.0–24.5)	18.8 (11.2–30.8)	17.3 (10.2–30.3)
sTfR mg/L, median (IQR)	1.26 (0.93–1.48)	2.35 (1.27–5.03)	2.18 (0.90–3.53)	1.39 (0.93–2.96) †
sTfR/log ferritin, median (IQR)	0.98 (0.77–1.31)	2.17 (0.67–5.47)	0.70 (0.55–2.23)	1.01 (0.71–2.30)
Anemic children meeting IDA criteria (%)	21/78 (26.9)	13/51 (25.5)	5/21 (23.8)	39/150 (26.0) §
Anemic children meeting AI criteria (%)	15/72 (20.8)	7/38 (18.4)	4/17 (23.5)	26/127 (20.5)
Anemic children meeting criteria for mixed AI with IDA (%)	5/72 (6.9)	4/38 (10.5)	3/17 (17.6)	12/127 (9.4)

MCV = Mean corpuscular volume; MCHC = Mean corpuscular hemoglobin concentration; IDA = iron-deficiency anemia, AI = anemia of inflammation; IQR = inter quartile range; Significant differences among age groups by x^2^ testing † *p* < 0.001; Overall significant association between anemia and IDA regardless of age, by x^2^ testing § *p* < 0.001.

## Data Availability

The data supporting the findings of this study are provided within the article.
